# Effects of Air Quality Index and meteorological factors on cardiovascular and cerebrovascular mortality in Huizhou, China: a time-series analysis using distributed lag nonlinear models

**DOI:** 10.3389/fpubh.2025.1694321

**Published:** 2025-12-01

**Authors:** Yangling Zhou, Qiaoer Lin, Binhua Qin, Zhiwen Zheng, Xulin Chen, Renzhao Pang, Jingrong Huang, Yuanbin He, Shiyuan Shen, Qili Chen, Liuqing Peng, Shuai Jiang, Caiming Li

**Affiliations:** 1 The First Huizhou Affiliated Hospital of Guangdong Medical University, Huizhou, China; 2 The First Clinical Medical College, Guangdong Medical University, Zhanjiang, China; 3 Department of Neurology, Huizhou First Hospital, Huizhou, China; 4 Huizhou City Emergency Warning Information Release Center, Huizhou, China; 5 Huizhou Meteorological Bureau, Huizhou, China

**Keywords:** cardiovascular diseases and cerebrovascular diseases, Air Quality Index, mortality risk, lag effects, meteorological factors

## Abstract

**Background:**

Air pollution exposure is recognized to exacerbate cardiovascular and cerebrovascular diseases (CVDs). This study investigated the association between the Air Quality Index (AQI) and CVD mortality in Huizhou, China, focusing on population-specific lag effects and interactions with meteorological factors.

**Methods:**

Daily CVD mortality data (2015–2021) from Huizhou were analyzed using distributed lag nonlinear models (DLNMs) and generalized additive models (GAMs) to assess AQI’s relationship with mortality, with subgroup analyses by gender and age. Poisson regression, based on interaction theory, clarified joint effects of AQI and meteorological factors.

**Results:**

AQI ≥ 80 was associated with increased CVD mortality risk with lag effects. Cumulative lag peaks varied: total population (2 days, RR = 1.00156, 95% CI: 1.00077–1.00235), males (3 days, RR = 1.00188), females (1 day, RR = 1.00130), ≥65 years (1 day, RR = 1.00098), and 65 years (3 days, RR = 1.00455). Mean wind speed showed an antagonistic interaction with AQI (IRR = 0.92749, 95% CI: 0.89575–0.96035), reducing risk, while mean humidity had a synergistic effect (IRR = 1.05124, 95% CI: 1.01395–1.08989), exacerbating risk.

**Conclusion:**

AQI is positively associated with CVD mortality, with lag effects differing by population. Wind speed mitigates, and humidity amplifies, AQI-related risks. These findings support targeted early warning systems and preventive strategies for CVDs.

## Highlights

- What is the relationship between AQI and cardiovascular and cerebrovascular disease mortality in Huizhou, and does this relationship exhibit lag effects? How do these lag effects vary across different populations?
  The study found a positive correlation between AQI and cardiovascular and cerebrovascular disease mortality in Huizhou. When AQI exceeds 80, the relative risk (RR) of mortality gradually increases, with significant lag effects. Specifically, the cumulative lag effect peaks at 2 days for the total population (RR = 1.00156, 95% CI: 1.00077–1.00235). For subgroups, males show the maximum cumulative lag effect at 3 days (RR = 1.00188), females at 1 day (RR = 1.00130), individuals aged ≥65 years at 1 day (RR = 1.00098), and those 65 years at 3 days (RR = 1.00455). These variations indicate population-specific differences in temporal responses to AQI exposure.
- Do meteorological factors (e.g., wind speed, humidity) interact with AQI to influence cardiovascular and cerebrovascular disease mortality in Huizhou? If so, what types of interactions are observed?
  Yes, significant interactions were identified. Mean wind speed exhibited an antagonistic effect with AQI: increased wind speed reduced the mortality risk associated with AQI, as indicated by an interaction relative risk (IRR) of 0.92749 (95% CI: 0.89575–0.96035), a relative excess risk of interaction (RERI) of −0.08008, and an attributable proportion (AP) of −0.07389. In contrast, mean humidity showed a synergistic effect with AQI: higher humidity exacerbated the mortality risk, with an IRR of 1.05124 (95% CI: 1.01395–1.08989), RERI of 0.04480, and AP of 0.04495. Temperature (mean and minimum) did not exhibit significant interactions with AQI.
- What implications do the study’s findings have for developing early warning systems and preventive measures for cardiovascular and cerebrovascular diseases in Huizhou?
  The findings support the development of stratified early warning systems tailored to population-specific vulnerabilities and meteorological conditions. Key implications include: (1) Establishing risk-based warning hierarchies (e.g., mild, moderate, severe) based on AQI thresholds (≥80) and combined meteorological factors (e.g., high humidity with low wind speed). (2) Implementing population-specific alerts: lowering thresholds for males due to higher exposure risks, emphasizing 3-day cumulative lag effects for individuals 65 years, and prioritizing winter alerts for those ≥65 years. (3) Integrating dynamic models that combine AQI and meteorological data (e.g., wind speed, humidity) to enhance prediction accuracy, thereby facilitating targeted preventive interventions for vulnerable groups.

## Introduction

Cardiovascular and cerebrovascular diseases (CVDs), as highly prevalent and lethal chronic diseases, have become an important factor threatening human health (1). In recent years, a large number of epidemiologic studies have shown that air quality is closely associated with the development of cardiovascular and cerebrovascular diseases (2, 3). Cohort studies have found that all-cause mortality increases with exposure time and pollutant concentration in both short- and long-term exposure to air pollutants (4), while the influence of meteorological factors on the incidence of cardiovascular and cerebrovascular diseases has also been biologically validated (5, 6).

However, most of the existing studies focus on the association between a single pollutant and cardiovascular and cerebrovascular diseases, which has obvious limitations. First, single pollutant indicators cannot fully reflect the air quality situation of the day; second, pollutants often exist in mixed forms in the actual environment, and meteorological conditions vary significantly in different regions, so it is difficult to reflect the combined effects of these complex factors by focusing on a single indicator.

The Air Quality Index (AQI), as a comprehensive evaluation index of air pollution, can visualize the potential health hazards of air quality and help to enhance public health awareness and self-management ability. However, due to regional and spatial differences in the distribution of air pollutants, studies in different regions have used different indicators, making it difficult to accurately assess the impact of local air quality on cardiovascular and cerebrovascular disease mortality. Meanwhile, due to the influence of global climate change, there are limitations in relying solely on air pollution indicators to construct an early warning system. Therefore, a comprehensive consideration of the interaction between pollution mixtures and city-specific meteorological factors is essential to reveal the relationship between air quality and cardiovascular and cerebrovascular disease mortality.

In this study, distributed lag nonlinear models (DLNMs) and generalized additive models (GAMs) were used to investigate the intrinsic relationship between AQI and cardiovascular and cerebrovascular disease mortality, and to analyze the differences in this relationship among different age and gender groups. The multiplicative and additive Poisson regression models based on the interaction theory were used to clarify the joint influence of AQI and meteorological factors on the mortality rate of cardiovascular and cerebrovascular diseases, with a view to providing scientific basis for the construction of a more improved and effective early warning system.

## Methods

### Study area

Huizhou is a city located in Guangdong Province in southern China, with a total area of 11,300 square kilometers and a resident population of about 6,073,400 people. Huizhou, located in South China, belongs to the subtropical monsoon climate, with small temperature differences between the four seasons. During the period from 1 January 2015 to 31 December 2021, the temperature ranged from 10 °C to 34 °C. The annual precipitation ranged from 1,770 to 2,200 mm, with high temperatures and rainy summers and dry winters.

### Death data collection

In this study, daily death data of permanent residents in Huizhou City were collected from 1 January 2015 to 31 December 2021 through the Huizhou City Population Death Information Registration and Management Department, and the data covered basic information such as gender, age, date of death, and specific cause of death. The screening of the population of cardiovascular and cerebrovascular disease deaths was based on the following criteria: (1) the main or underlying cause of death was consistent with the ICD-10 codes I51.6 and I67.9 categories; and (2) belonging to the permanent residents within Huizhou City.

### Meteorological data collection

The meteorological data used in this study were obtained from the Huizhou Meteorological Bureau, which collected the meteorological data of Huizhou City for the period from 1 January 2015 to 31 December 2021, including the daily maximum temperature (°C), daily minimum temperature (°C) and daily average atmospheric pressure (Pa), daily average wind speed (m/s), and so on.

### Air pollutant data collection

The air pollution data of Huizhou City from 2015 to 2021, including PM_2.5_, PM_10_, NO_2_, SO_2_, CO, and O_3_, were collected from the official website of the Weather Post,^1^ which was sourced from the National Environmental Monitoring General Station.

Calculation of AQI (7)


$${\text{IAQI}}_{P}=\frac{\mathrm{IAQ}I_{\mathrm{Hi}}-\text{IAQI}L_{0}}{BP_{\mathrm{Hi}}}-BP_{L0}(C_{P}-BP_{L0})+\text{IAQI}L_{0}$$

$$\mathrm{AQI}=\max\{\mathrm{AQ}I_{1},\mathrm{AQ}I_{2},\mathrm{AQ}I_{3},\cdots \cdots {\mathrm{AQI}}_{P}\}$$

${\text{IAQI}}_{P}$ : that is, Air Quality Sub-Index, the Air Quality Index for a single pollutant;

$C_{P}$ : the mass concentration value of pollutant item P;

$BP_{\mathrm{Hi}}$ : the high end of the pollutant concentration limit similar to $C_{P}$ ;

$BP_{L0}$ : the low value of pollutant concentration limit similar to $C_{P}$ ;

$\mathrm{IAQ}I_{\mathrm{Hi}}$ : Air Quality Sub-Index corresponding to $BP_{\mathrm{Hi}}$ ;

$\text{IAQI}L_{0}$ : the Air Quality Sub-Index corresponding to $BP_{L0}$ ;

During the study period, the AQI in Huizhou ranged from 11 to 180. The total number of days during the study period was 2,557 according to the Ambient Air Quality Standards (GB3095-2012). Among them, the number of days with excellent air quality was 1,613, good air quality 932, mild pollution 11, moderate pollution 1, and no days with severe or heavy pollution. The main exceeding pollutants were ozone and fine particulate matter.

### Statistical analysis

Using a time-series study, this research explores the impact of AQI on cardiovascular and cerebrovascular disease mortality in Huizhou, providing more evidence for improving early warning systems. We adopted a two-step approach to evaluate the association between AQI and cardiovascular and cerebrovascular disease mortality. Briefly, in the first stage, this study explored the exposure-response relationship between AQI and cardiovascular and cerebrovascular disease mortality, followed by an analysis of the interaction between AQI and meteorological factors (e.g., temperature, humidity). The two steps are described in more detail below.

### Phase 1: To establish the relationship between cardiovascular and cerebrovascular disease mortality and AQI exposure

Previous studies have shown that daily cardiovascular and cerebrovascular disease deaths, since they are rare events, can be approximated by a Poisson distribution. Additionally, evidence indicates that the AQI-disease exposure–response relationship is inherently nonlinear with lagged effects. Therefore, our study employed distributed lag nonlinear models (DLNMs) in conjunction with generalized additive models (GAMs) to capture both nonlinear and lag effects simultaneously. Given the substantial variability in baseline concentrations across pollutants, we used 10% of each pollutant’s annual average concentration as the unit for concentration change in our model. The Akaike information criterion (AIC) was used to select the optimal degrees of freedom (df). The distributed lag nonlinear models (DLNMs) quantifying the relationship between AQI exposure and cardiovascular and cerebrovascular disease mortality were specified as follows:


$$\begin{array}{l} Y_{t}\sim \text{Poisson}(\mathrm{\mu t})\log(\mathrm{\mu t})=\alpha +\mathrm{cb}(\chi_{t,l})+\mathrm{ns}(\mathrm{Tim}e_{t,},\mathrm{df}=7)\\ +\mathrm{cb}(\text{pressur}e_{t,l},\mathrm{df}=3)+\mathrm{cb}(\text{mean wind velocit}y_{t,l},\mathrm{df}=3)\\ +{\text{ηDOW}}_{t}+{\text{γHoliday}}_{t} \end{array}$$

where $Y_{t}$ is the daily number of cardiovascular and cerebrovascular deaths on day t ; α is the intercept of the model; $\mathrm{cb}(\chi_{t,\iota })$ is a cross-basis function to model the nonlinear lagged effects of the daily independent variables; $\chi_{t}$ are the AQI, mean temperature, and relative humidity, respectively; l is the lag of $\chi_{t}$ ; and ns is the natural cubic spline function from the R-package “ dlnm ”. To control for long-term trends and seasonality, we used a natural cubic spline function with seven degrees of freedom per year ( df ) and an indicator for working days (DOW).

The generalized additive model (GAM) for the relationship between AQI exposure and cardiovascular and cerebrovascular disease mortality was specified as follows:


$$\begin{array}{l} \log[E(Y_{t})]=\mathrm{cb}({\mathrm{TI}}_{t},\mathrm{lag})+\mathrm{ns}({\text{time}}_{t},\mathrm{df}=7*7)\\ +\mathrm{ns}({\mathrm{Rh}}_{t},\mathrm{df}=3)+\mathrm{ns}({\mathrm{WS}}_{t},\mathrm{df}=3)+\mathrm{DOW}+\text{Holiday}+\alpha \end{array}$$

where Y, total cardiovascular and cerebrovascular disease deaths on day t; cb , cross-basis function; lag, lag time; ns, natural cubic spline function; df , degree of freedom parameter; *α*-intercept; TIt , AQI on day t; Rh , mean daily relative humidity; WS, mean daily wind speed. The degrees of freedom for relative humidity and wind speed were both set to 3; time, time variable, the degrees of freedom were chosen as 7 times the number of years according to the literature; DOW, day of the week, which was introduced into the model as a dummy variable.

### Phase 2: assessment of possible interactions between temperature, humidity, and AQI values

Based on the results of the first stage, temperature, wind speed, and humidity were categorized into two groups (low/high) according to their turning points, while AQI was classified into two types based on national standards (≤100 as good; >100 as polluted). In summary, the interaction model is as follows:Using the Poisson regression model, based on the multiplicative and additive models of interaction theory, the interaction coefficients can be obtained, and the interaction relative risk (IRR), relative excess risk due to interaction (RERI), and attributable proportion due to interaction (AP) can be calculated. The calculation methods are as follows:


$$Y_{t}\sim \text{Poisson}(\mu_{t})=\alpha +T(\mathrm{orH})+\mathrm{AQI}+\mathrm{AQI}:T(\mathrm{orH})$$

$$\mathrm{IRR}={\mathrm{RR}}_{11}/({\mathrm{RR}}_{01}*{\mathrm{RR}}_{10})$$

$$\text{RERI}={\mathrm{RR}}_{11}-{\mathrm{RR}}_{10}-{\mathrm{RR}}_{01}+1$$

$$\mathrm{AP}=\text{RERI}/{\text{OR}}_{11}$$

If IRR is greater than 1 or RERI is greater than 0, it indicates a synergistic effect between meteorological factors and AQI; if IRR is less than 1 or RERI is less than 0, an antagonistic effect exists; otherwise, when IRR equals 1 or RERI equals 0, it suggests no interaction. Additionally, AP refers to the attributable proportion caused by the interaction. For all statistical tests, a two-sided *p*-value 0.05 was considered statistically significant. A 95% confidence interval (CI) of AP including 0 indicates no additive interaction, while a 95% CI of AP not including 0 indicates the presence of additive interaction.

## Results

### Analysis of the number of deaths from cardiovascular and cerebrovascular diseases and meteorological factors in Huizhou City

Based on our preliminary statistics on different AQI values, corresponding cardiovascular and cerebrovascular disease deaths, meteorological indicators, and pollutants. From 2015 to 2021, the number of cardiovascular and cerebrovascular disease deaths among permanent residents in Huizhou was 66,154, with males accounting for 53.30% and females 46.70%. The average daily total deaths were 24.00, including 13.80 daily deaths in males and 12.10 in females.

For meteorological indicators, the medians (P50) of average air pressure, temperature, wind speed, and humidity were 1000.7 Pa, 24.10 °C, 2.000 m/s, and 78.00%, respectively. For air pollutants, the medians (P50) of NO₂, O₃, PM_2.5_, PM_10_, SO₂, and CO were 20.00 μg/m^3^, 58.00 μg/m^3^, 22.00 μg/m^3^, 42.00 μg/m^3^, 7.000 μg/m^3^, and 0.6700 mg/m^3^, respectively. Meanwhile, the median and mean of AQI were 44.00 and 45.93, respectively.

Detailed characteristics of the study population, meteorological data, and pollutant parameters are presented in Table 1.

**Table 1:** Descriptive statistics of all deaths from cardiovascular and cerebrovascular diseases, meteorological indicators and pollutants in Huizhou city during the period 2015–2021.

Items	Clusters	Frequency distribution					
		Min	Q1 (*p*_(25)_)	Median (*p*_(50)_)	Q3 (*p*_(75)_)	Max (*p*_(100)_)	Average value
Deaths from cardiovascular diseases	Total (persons)	7.00	20.00	24.00	30.00	75.00	25.80
	Male (persons)	2.00	10.00	13.00	16.00	46.00	13.80
	Women (persons)	1.00	9.00	11.00	15.00	39.00	12.10
Meteorological indicators	Average air pressure (Pa)	982.3	995.7	1000.7	1005.5	1022.5	1000.7
	Average temperature (°C)	2.800	18.80	24.10	27.70	32.10	22.92
	Average wind speed (m/s)	0.400	1.600	2.000	2.600	9.300	2.171
	Humidity (%)	26.00	70.00	78.00	85.00	100.00	76.30
Pollutants	NO_2 (_μg /m3)	3.00	15.00	20.00	26.00	71.00	21.56
	O_3 (_μg/m3)	7.00	42.00	58.00	77.00	196.00	61.15
	PM_2.5 (_μg/m3)	1.00	14.00	22.00	32.00	142.00	24.29
	PM_10 (_μg/m3)	5.00	29.00	42.00	57.00	189.00	45.06
	SO_2 (_μg/m3)	3.000	6.000	7.000	9.000	23.000	7.623
	CO (μg/m3)	0.3600	0.5600	0.6700	0.8000	1.5200	0.6987
	AQI	11.00	32.00	44.00	57.00	180.00	45.93

### The relationship between AQI and cardiovascular and cerebrovascular disease mortality in Huizhou

**Figure 1:**
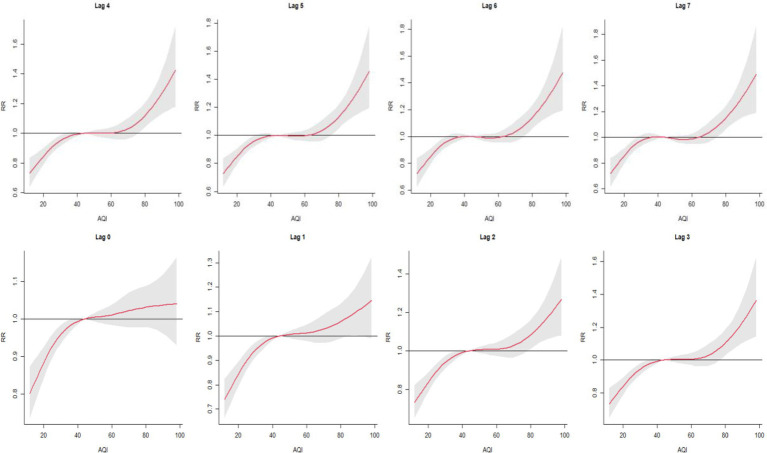
Cumulative lag (0–7 days) association between AQI and cardiovascular mortality in Huizhou City (2015–2021) as estimated by distributed lag nonlinear models (DLNMs).

As shown in Figure 1, RR values gradually increased as AQI exceeded 80, indicating a positive correlation between AQI and cardiovascular mortality. The lag effect of AQI on total CVD mortality was as follows: the cumulative mortality effect peaked at a lag of 2 days, with an RR of 1.00156 (95% CI: 1.001–1.002, *p*  0.05). See Table 2 and Figure 1 (Note: Data are presented with five decimal places. CI = confidence interval).

**Table 2:** Relative risk of cardiovascular mortality associated with AQI in Huizhou City (2015–2021).

Lag (d)	Total population	Males	Females	≥65 years	65 years
Lag0	1.00096 (1.00031 ~ 1.00161)	1.00093 (1.00008 ~ 1.00178)	1.00088 (0.99999 ~ 1.00176)	1.00053 (0.99982 to 1.00125)	1.00320 (1.00172 ~ 1.00470)
Lag1	1.00143 (1.00078 ~ 1.00208)	1.00148 (1.00064 ~ 1.00233)	1.00125 (1.00036 ~ 1.00213)	1.00107 (1.00036 ~ 1.00179)	1.00301 (1.00152 ~ 1.00451)
Lag2	1.00084 (1.00019 ~ 1.00149)	1.00108 (1.00023 ~ 1.00192)	1.00046 (0.99958 ~ 1.00135)	1.00039 (0.99968 to 1.00111)	1.00298 (1.00149 ~ 1.00448)
Lag3	1.00048 (0.99983 ~ 1.00114)	1.00105 (1.00020 ~ 1.00189)	0.99975 (0.99886 ~ 1.00064)	1.00013 (0.99941 to 1.00084)	1.00187 (1.00037 ~ 1.00337)
Lag4	1.00019 (0.99953 to 1.00084)	1.00046 (0.99962 to 1.00131)	0.99978 (0.99890 ~ 1.00068)	1.00007 (0.99936 ~ 1.00078)	1.00045 (0.99895 ~ 1.00196)
Lag5	0.99988 (0.99922 ~ 1.00054)	0.99988 (0.99904 ~ 1.00073)	0.99978 (0.99889 ~ 1.00067)	0.99973 (0.99902 to 1.00045)	1.00064 (0.99913 to 1.00214)
Lag6	0.99997 (0.99932 to 1.00063)	0.99997 (0.99912 ~ 1.00082)	0.99988 (0.99899 ~ 1.00076)	0.99984 (0.99912–1.00055)	1.00059 (0.99909 ~ 1.00210)
Lag7	0.99983 (0.99918 ~ 1.00049)	0.99985 (0.99900 ~ 1.00070)	0.99972 (0.99883 ~ 1.00061)	0.99993 (0.99921–1.00064)	0.99968 (0.99817 ~ 1.00118)
Lag01	1.00146 (1.00074 ~ 1.00219)	1.00147 (1.00053 ~ 1.00240)	1.00130 (1.00032 ~ 1.00228)	1.00098 (0.00019 to 1.00177)	1.00379 (1.00214 ~ 1.00544)
Lag02	1.00156 (1.00077 ~ 1.00235)	1.00167 (1.00065 ~ 1.00269)	1.00125 (1.00018 ~ 1.00232)	1.00095 (1.00009 ~ 1.00181)	1.00439 (1.00439 ~ 1.00618)
Lag03	1.00156 (1.00071 ~ 1.00240)	1.00188 (1.00079 ~ 1.00297)	1.00098 (0.99983 ~ 1.00213)	1.00087 (0.99995 ~ 1.00180)	1.00455 (1.00264 ~ 1.00647)
Lag04	1.00149 (1.00059 ~ 1.00240)	1.00188 (1.00071 ~ 1.00304)	1.00082 (0.99960 to 1.00205)	1.00082 (0.99983 to 1.00181)	1.00429 (1.00225 ~ 1.00633)
Lag05	1.00135 (1.00039 ~ 1.00231)	1.00170 (1.00047 ~ 1.00294)	1.00069 (0.99940 to 1.00199)	1.00066 (0.99961 ~ 1.00171)	1.00420 (1.00205 ~ 1.00636)
Lag06	1.00128 (1.00026 ~ 1.00229)	1.00160 (1.00031 ~ 1.00290)	1.00062 (0.99925 ~ 1.00199)	1.00056 (0.99945 to 1.00167)	1.00418 (1.00191 ~ 1.00645)
Lag07	1.00120 (1.00013 ~ 1.00226)	1.00152 (1.00015 ~ 1.00288)	1.00052 (0.99908 ~ 1.00197)	1.00052 (0.99936 ~ 1.00169)	1.00396 (1.00158 ~ 1.00634)

LagX represents a single-day lagged X-day effect; Lag0X represents a cumulative lagged X-day effect.

Lag0X represents the cumulative lag X days effect.

### Effects of Huizhou’s AQI on cardiovascular and cerebrovascular mortality in male and female populations

**Figure 2:**
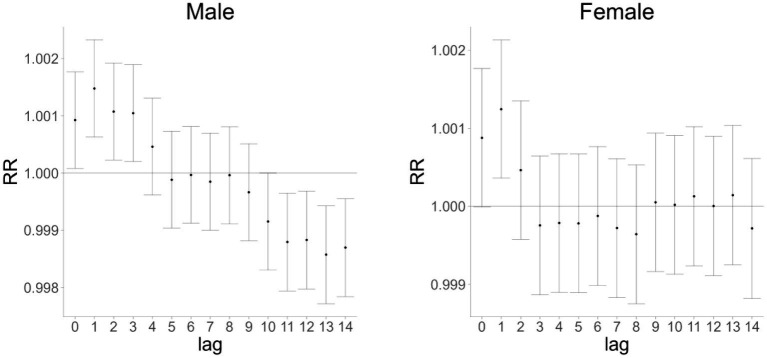
Single-day lag effect of AQI on cardiovascular and cerebrovascular mortality in male and female populations in Huizhou City (2015–2021).

**Figure 3:**
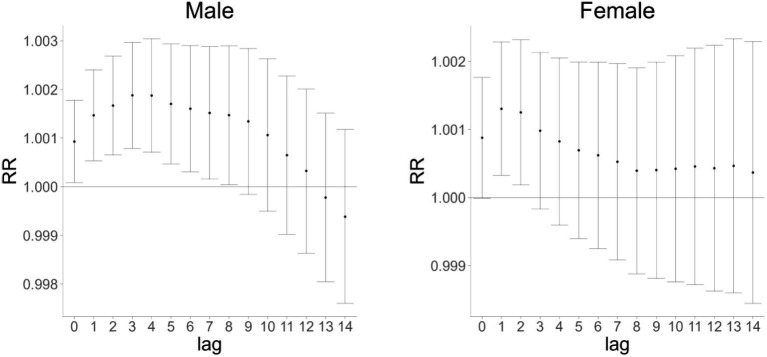
Cumulative lag effect of AQI on cardiovascular mortality in male and female populations in Huizhou City (2015–2021).

Further exploration of the lag effects of AQI on cardiovascular and cerebrovascular disease mortality in male and female populations in Huizhou from 2015 to 2021 revealed that AQI exerted lag effects on mortality in both genders (Figure 2). The maximum impact of AQI on cardiovascular and cerebrovascular disease mortality in males and females occurred at a single-day lag of 1 day, with relative risk (RR) values of 1.0014 [95% confidence interval (CI): 1.001–1.002] and 1.0012 (95% CI: 1.000–1.002), respectively. As shown in Figure 3, the cumulative lag effect of AQI on cardiovascular and cerebrovascular deaths peaked at 3 days in the male population and at 1 day in the female population, indicating gender-specific differences in temporal response patterns (Figures 2, 3 are cited herein to support this section).

### Effects of Huizhou’s AQI on cardiovascular and cerebrovascular mortality in populations aged ≥65 and 65 years

**Figure 4:**
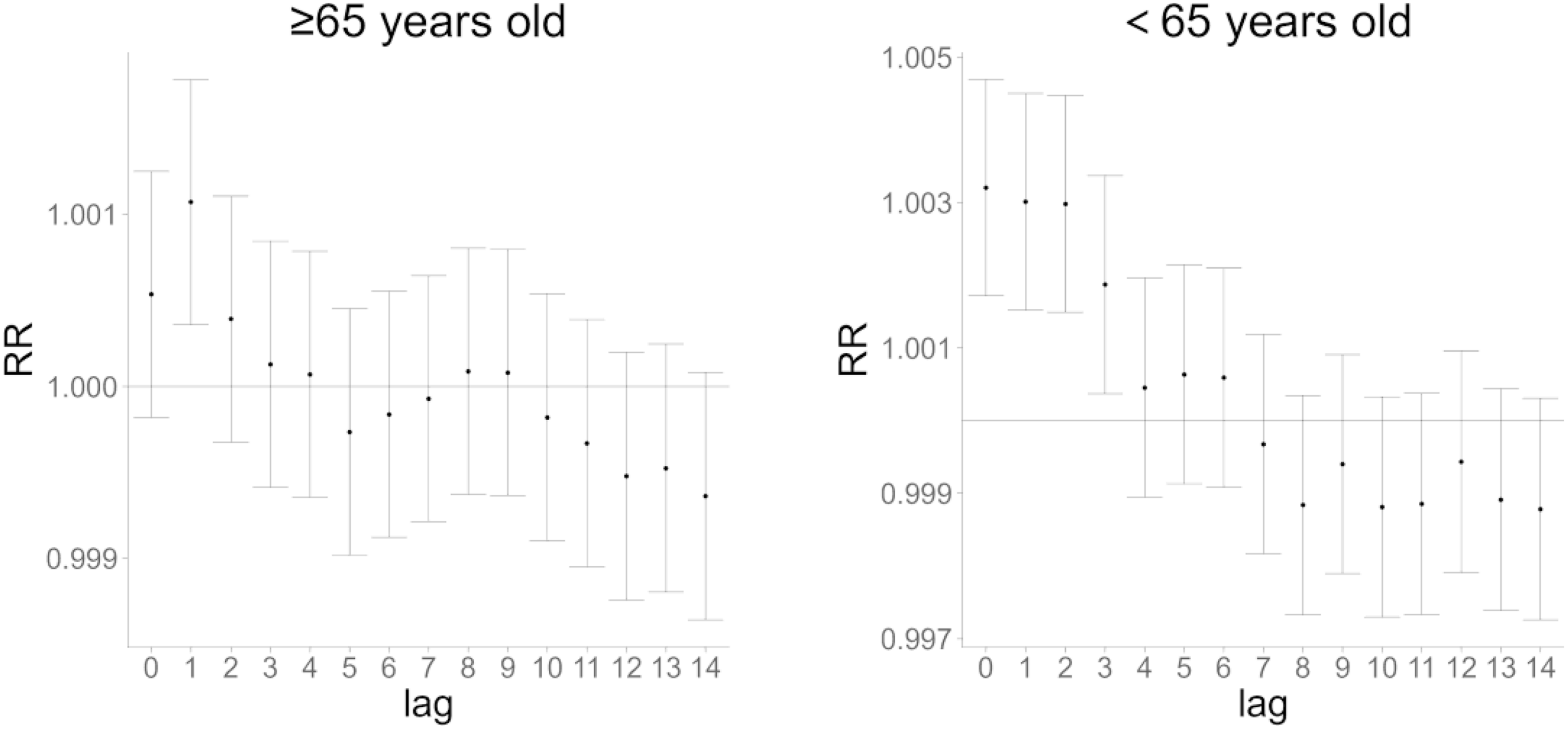
Single-day lag effect of AQI on cardiovascular and cerebrovascular mortality in age subgroups (≥65 vs. 65 years) in Huizhou City (2015–2021).

**Figure 5:**
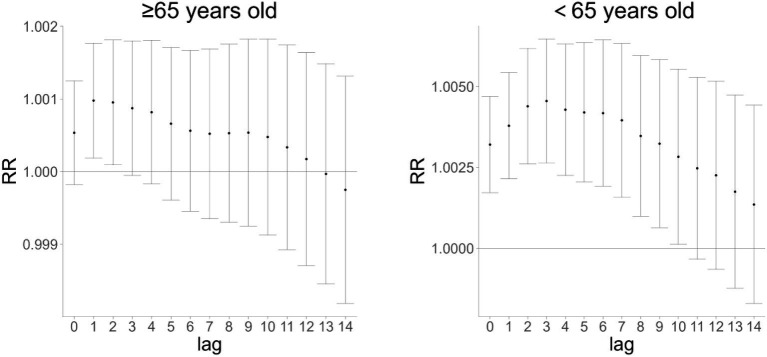
Cumulative lag effect of AQI on cardiovascular and cerebrovascular mortality in age subgroups (≥65 vs. 65 years) in Huizhou City (2015–2021).

To investigate the impact of AQI on cardiovascular and cerebrovascular disease deaths across different age groups, Figures 4, 5 show that AQI exerted lag effects on mortality in both the ≥65 years and 65 years age groups. For single-day lag effects, the maximum impact of AQI on cardiovascular and cerebrovascular deaths occurred at lag 1 day in the ≥65 years group and at lag 0 days in the 65 years group, with relative risk (RR) values of 1.00107 (95% confidence interval [CI]: 1.00036–1.00179) and 1.00321 (95% CI: 1.00172–1.00470), respectively. For cumulative lag effects, the peak impacts were observed at cumulative lag 1 day in the ≥65 years group (RR = 1.00098, 95% CI: 1.00019–1.00177) and at cumulative lag 3 days in the 65 years group (RR = 1.00455, 95% CI: 1.00264–1.00647) (Figures 4, 5 are cited herein to support this section).

### Interaction analysis

Table 3 presents the effects of interaction terms between temperature, wind speed, humidity, and AQI in the overall population. As shown, the interactions between mean temperature/lowest temperature and AQI were not significant, indicating that temperature and AQI had no obvious combined effect on the risk of cardiovascular and cerebrovascular mortality. In contrast, the interaction between mean wind speed and AQI was significant and negative, suggesting that increased wind speed may reduce the risk of cardiovascular and cerebrovascular death. Conversely, the interaction between mean humidity and AQI was significant and positive, implying that higher humidity may exacerbate the risk associated with AQI exposure.

**Table 3:** Interaction effects of temperature, wind speed, humidity, and AQI on cardiovascular disease mortality risk.

Variables	IRR (95% CI)	RERI (95% CI)	AP (95% CI)
Mean temperature	0.98570 (0.95125–1.02140)	−0.02060 (−0.05424–0.01304)	−0.02344 (−0.06173–0.01485)
Minimum temperature	0.98205 (0.94733–1.01804)	−0.02266 (−0.05646–0.01114)	−0.02623 (−0.06538–0.01291)
Mean wind speed	0.92749* (0.89575–0.96035)	−0.08008* (−0.11771–0.04244)	−0.07389* (−0.10870–0.03907)
Mean humidity	1.05124* (1.01395–1.08989)	0.04480* (0.00956–0.08004)	0.04495* (0.00971–0.08018)

* Statistically significant interaction (*p* 0.05, 95% CI does not include 0 for RERI/AP or 1 for IRR).

IRR: Interaction relative risk (IRR > 1 = synergism; IRR 1 = antagonism).

RERI: Relative excess risk of interaction (RERI > 0 = synergism; RERI 0 = antagonism).

AP: Attributable proportion due to interaction.

## Discussion

This study investigated the association between Air Quality Index (AQI) and cardiovascular-cerebrovascular mortality in Huizhou during 2015–2021, as well as the joint effects of meteorological factors and AQI on mortality risk, revealing the complexity and lagged nature of AQI’s impact on cardiovascular-cerebrovascular health.

Significant positive correlation was observed between AQI and cardiovascular-cerebrovascular mortality, with risk escalating when AQI ≥ 80 and exhibiting a 1–3-day lag effect. This finding aligns with global studies showing that air pollutants (SO₂, NO₂, CO, O₃, PM₂.₅, PM₁₀) significantly impact cardiovascular-cerebrovascular diseases (8). Short-term exposure to high AQI exacerbates risks through mechanisms such as inducing inflammatory responses, oxidative stress, and vascular endothelial dysfunction (2, 3, 9). AQI may also indirectly increase risk via effects on mental health (e.g., anxiety, depression) (10). Notably, despite Huizhou’s favorable air quality (only 12/2557 days with mild or worse pollution), minor AQI fluctuations still threatened vulnerable populations, suggesting a need to optimize current air quality standards, particularly for key pollutants like ozone and PM₂.₅ (11).

This study revealed significant differences in the cumulative lag effects of AQI on cardiovascular-cerebrovascular mortality across genders and age groups, with males and individuals 65 years exhibiting higher relative risk (RR) values and statistically significant differences. These findings suggest that differences in gender-specific physiological characteristics and pollutant sensitivity may underlie the divergent temporal responses. Gender disparities may be attributed to males’ higher outdoor exposure, smoking rates, and occupational pollutant exposure (1), as well as hormonal influences. Estrogen has been shown to mitigate oxidative stress and activate antioxidant and longevity-related genes, conferring cardiovascular protection in females (12). For younger populations, prolonged pollutant exposure due to higher daily activity levels likely amplifies risks during the cumulative lag phase (13). This finding highlights the necessity of implementing stratified early warning systems and provides a theoretical basis for developing targeted health interventions, emphasizing the need for precision-tailored strategies to address population-specific vulnerabilities.

Findings from interaction analysis showed that increased wind speed mitigated the risk of cardiovascular and cerebrovascular mortality associated with AQI (IRR = 0.9275), whereas higher humidity exacerbated this risk (IRR = 1.0512). Wind speed likely reduces exposure by accelerating pollutant dispersion (14), while high humidity may promote the formation, accumulation, and secondary reactions of certain pollutants by increasing atmospheric water vapor content, thereby elevating AQI values (15). Although low temperature has been shown to influence cardiovascular and cerebrovascular mortality—particularly among individuals ≥60 years (16)—Huizhou’s subtropical monsoon climate (mean temperature range: 10 °C to 34 °C) and low frequency of extremely low-temperature days, combined with nonsignificant interactions between minimum temperature and AQI, suggest that low temperature exerts a smaller impact on mortality compared to wind speed and humidity in this region. These findings highlight the need to integrate multiple meteorological and air quality factors when assessing cardiovascular and cerebrovascular disease risk, providing a comprehensive basis for formulating public health prevention and control strategies.

### Stratified early warning mechanism based on lag effects

#### Risk-level based warning hierarchy

Warning levels are classified according to AQI values and combinations of meteorological factors:

- Mild warning: AQI 80–100 with high humidity and low wind speed.
- Moderate warning: AQI 101–150 with exceeding standards of key pollutant concentrations and unfavorable meteorological conditions for pollutant dispersion.
- Severe warning: AQI > 150 or superposition of multiple adverse factors.

#### Population-specific differential warning

- Gender stratification: The warning threshold for males is reduced by 10% due to their higher outdoor exposure, smoking rates, and occupational exposure, requiring earlier intervention.
- Age stratification:
- 65 years: Focus on risks at a cumulative lag of 3 days, with warning messages emphasizing the risk of “prolonged exposure from daily activities.”
- ≥65 years: Issue cardiovascular risk alerts during winter (low temperature) even if AQI thresholds are unmet, especially when combined with high AQI.
- Vulnerable groups: Use electronic health records to flag individuals with hypertension, diabetes, cardiovascular diseases, or other underlying conditions for precise delivery of warning information.

#### Precision prediction

Integrate meteorological and environmental protection data to construct dynamic risk assessment models. Machine learning is applied to predict the health impacts of AQI changes (17, 18), enhancing the accuracy of the early warning system.

The innovation of this study lies in using the Air Quality Index (AQI) instead of single air pollutants to comprehensively demonstrate the impact of air pollution on cardiovascular and cerebrovascular mortality, which more closely simulates real-world exposure scenarios. As a relatively mature air quality indicator, AQI is supported by long-term monitoring data. Its standardized and visualized design facilitates more effective early warning and prevention (19). Additionally, the analysis of interaction effects between meteorological factors and AQI provides a more comprehensive understanding of how air quality influences cardiovascular and cerebrovascular mortality, offering new insights for developing more precise early warning systems and prevention strategies.

This study also has certain limitations.

1. Limitations in original data processing:

Confounding variables such as individual behaviors (e.g., smoking, physical activity) and genetic factors (20), as well as socioeconomic variables including education level, income, and medical resources (21), were not controlled for. Additionally, while AQI and air pollutant data in this study were all obtained from official monitoring stations covering the entire administrative area of Huizhou, details regarding data quality control (e.g., outlier handling, completeness verification) and the rationality of monitoring station distribution were not elaborated. Furthermore, unique variables specific to the COVID-19 pandemic in Huizhou—such as air pollution characteristics, population access to healthcare, and health-seeking behaviors of patients with cardiovascular and cerebrovascular diseases—were not controlled either, nor was specialized validation conducted on the data from the pandemic period (22).

2 Limitations in the extrapolation of study results:

- Relying solely on the Air Quality Index (AQI) instead of actual pollutant concentrations lacks precision in identifying key pollutant drivers. It is thus impossible to accurately determine which pollutant has the most significant impact on cardiovascular health, making it difficult to extend the results to regions with different pollutant profiles.
- Since the AQI was calculated based on Chinese standards, the results cannot be directly applied to regions adopting other AQI calculation systems.
- The data are limited to a single city (where days with excellent or good AQI account for 99.5%), so caution is required when extrapolating the conclusions.

Future research on this study can be advanced in the following aspects:

1. Adopt the multiple exposure distributed lag model (23) to consider the combined effects of multiple pollutants while distinguishing their independent effects, thereby improving the accuracy of risk attribution.
2. Optimize interaction analysis methods: Integrate AQI, meteorological factors, and their lag effects into a unified continuous modeling framework; or Adopt spatial and spatiotemporal variants of the distributed lag nonlinear model (DLNM) and generalized additive model (GAM) (24) to break through the methodological limitations of single-region studies. This will enable more accurate capture of the “pollution-health” association in air pollution epidemiological studies.
3. Expand to multicenter cohort studies. Specifically, integrate subgroup analysis results from the current study to supplement investigations into pollutant-specific mechanisms underlying cerebrovascular damage. Furthermore, link the higher relative risk (RR) observed in the younger population to their characteristics of greater physical activity and longer exposure duration, which will explain the mechanistic rationality of this elevated risk and enhance the persuasiveness of conclusions. Additionally, combine biomarkers (e.g., inflammatory factors) to explore the molecular mechanisms underlying AQI-induced pathogenesis (25), and evaluate the practical effectiveness of prevention and control measures through intervention experiments.

## Data Availability

The original contributions presented in the study are included in the article/supplementary material, further inquiries can be directed to the corresponding author.

## Footnotes

^1^ https://www.tianqihoubao.com/
